# Constraining the lifespans of early animals of the Ediacaran

**DOI:** 10.1098/rsbl.2025.0348

**Published:** 2025-08-20

**Authors:** Emily G. Mitchell, Alavya Dhungana

**Affiliations:** ^1^ Department of Zoology, University of Cambridge, Cambridge, UK; ^2^ University Museum of Zoology, Cambridge, CB2 3EJ, UK; ^3^ Department of Earth Sciences, Durham University, Durham, UK

**Keywords:** Ediacaran, metabolic theory of ecology, lifespan, evolutionary rates

## Abstract

Lifespans fundamentally impact life-history traits. These traits influence the tempo of biological cycles such as nutrient cycling and macro-evolutionary patterns over geological time. Yet, the lifespans of the first animals, found during the Ediacaran Period (approx. 580–539 Ma), are not well constrained, limiting our understanding of ecological and evolutionary change of early animals. In this study, we use the metabolic theory of ecology to estimate the maximum lifespans and evolutionary rates of 10 key Ediacaran taxa, constraining maximum lifespan variation for different environmental temperatures and modularity. We find a large range of different maximum lifespans for Ediacaran taxa (0.53–30.2 years), with longer lifespans in colder environments and for modular organisms (up to 40.4 years). Evolutionary rates were most impacted by environmental temperature, with the fastest evolutionary rates found in small, warm-water taxa. Ediacaran organisms pre-date macro-predation and so do not suffer this key downside of small body size. Therefore, these small, warm-water taxa kept the advantages of these higher evolutionary rates, without the predatory downside. The release from predation coupled to these fast evolutionary rates could help to explain the large morphological and taxonomic diversity found in the shallow-water Ediacaran assemblages, in contrast to the colder, deep-water assemblages.

## 1. Introduction

Life-history traits of animals are central to understanding their evolution as they directly impact organism fitness. Lifespans are a crucial trait as they often reflect trade-offs between survival, reproductive and growth traits [1]. Yet relatively little is known about lifespans of the earliest animals found during the Ediacaran time period [2,3], due to the limited preservation of phylum-level homologies [4–6]. The majority of these animals are non-biomineralizing, so there are limited methods for inferring lifespan such as can be done with bone or shell growth [7].

Fortunately, lifespans can be inferred using the metabolic theory of ecology (MTE); this framework can infer an organism’s metabolic rate given its body mass and habitat temperature. The MTE predicts the relationship of organism-level traits of metabolic rates to ecological traits such as population growth, carrying capacity and biodiversity [8–10]. These predictions match empirical observations of lifespans and growth rates across a range of taxa including unicellular plankton, bacteria, plants, invertebrates such as corals, and vertebrates such as fish, as well as large-scale patterns in current global diversity patterns such as the latitudinal diversity gradient [8,10–14]. These scaling laws are suggested to hold due to metabolic rates changing because smaller organisms need more energy (per unit biomass) to transfer nutrients around their body than larger organisms due to their increased surface area to volume ratios, although the underlying mechanisms for the MTE scaling relationships are debated [8,15–17]. Besides body mass, temperature is key to the scaling laws as this variable fundamentally changes the rate of physiological and biochemical reactions [10].

In this study, we will use the MTE to predict the lifespans and relative evolutionary rates for key Ediacaran taxa, across a range of different environmental temperatures. Ediacaran taxa such as *Arborea* have been suggested to be colonial [18], whereby individual organisms are formed by modular units which are physically connected and so share resources such as food [19]. There is also debate around the Ediacaran clade rangeomorphs such as *Charnia, *as to whether they are are colonial cnidarians [20,21]. Therefore, we also consider the impact of modularity, namely, colonial integration, on maximum lifespans and relative evolutionary rates.

## 2. Methods

In order to use the MTE to calculate lifespan and evolutionary rates, we need to calculate the biomass of our Ediacaran organisms and parametrize equations (2.1) and (2.2).

### (a) Model parameters

The temperature-corrected metabolic rate $\overset{-}{B}$ for a given organism is given by:


(2.1) $$\overset{-}{B\;}=b_{0}M^{\beta }e^{\frac{-E}{kT}},$$

where *β* is the mass scaling component predicted by MTE and the metabolic scaling theory as *−*1/4 for the temperature corrected metabolic rate, *M* is the wet body mass in grams of the organism, *E* is the activation energy, *b_0_* is the taxa dependent constant which for invertebrates is ln(*b_0_*) = 17.17 [10]. The activation energy *E* varies between 0.2 and 1.2 eV, but in an MTE context has consistently been observed as 0.65 eV [22,23], where 1 eV = 1.602 × 10^−19^ J, *k* is the Boltzmann constant 8.62 × 10−5 eV K^−1^, *M* is mass in grams and *T* is the absolute temperature.

The mass scaling component has been repeatedly validated against empirical data [22,23], consistently finding these relationships across all kingdoms of life, in different environments and across 50 orders of magnitude of body size, from bacteria to large whales [10,13,24,25], including deep sea taxa [26]. The largest deviations tend to include endothermic organisms, likely because they do not take on the ambient temperature of their environment such that birds, mammals and some fish have greater deviations from the MTE predictions [10,27]. The wide applicability of the MTE across phyla and body sizes is focused on unitary organisms, with only a limited number of modular or colonial organisms included in most studies. This focus on unitary organisms limits our understanding of the extent to which modularity, where organisms are formed of discrete, interconnected units, differs from unitary organisms [28]. While less understood, there is more variation of the metabolic scaling factor among modular organisms, with a mean value for modular organisms of −0.21 versus 0.35 for the measured unitary organisms, albeit with a range of different methods used to derive them [29].

The lifespan *Z* is inversely proportional to the metabolic rate [12]:

(2.2) $$\frac{1}{Z}=f\;\overset{-}{B\;}=f\;b_{0}M^{\beta }e^{\frac{-E}{kT}},$$

where *f* is predicted to be −0.65 eV for invertebrates and verified within the observed values [12]. The evolutionary rate of an organism is proportional to the metabolic rate and has been verified using the model group foraminifera using speciation rates derived from fossil and extant foraminifera [30]. Therefore, by calculating the predicted metabolic rate of an organism, the evolutionary rates can also be inferred.

### (b) Ediacaran inputs

Calculations of lifespan and evolutionary rates were performed in R (v. 4.3.2) with code and data available in the electronic supplementary material. The two key variables within the MTE are the temperature of the environment and the mass of the organism in grams. For Ediacaran organisms, we cannot measure biomass directly, so we have estimated this variable. Sea temperatures in the Ediacaran are thought to be similar to present day [31,32], so that the environmental temperature that Ediacaran organisms live in can be inferred using known Ediacaran biogeography [33] and knowledge of how modern sea temperatures change with latitude and depth. Ediacaran palaeography is hard to resolve due in part to the complex palaeomagnetism during this time [34] (and details within [35]), but recent reconstructions have Newfoundland and Charnwood Forest at high latitudes, Namibia, at mid-to-high latitudes and the White Sea and Ediacaran member at low latitudes [33,35] so for temperature inference we take latitudes of −64.2345 for Newfoundland and Charnwood taxa, −53.7376 for the Namibian taxa and 8.85673 [35] for the White Sea taxa, while acknowledging that these values do not capture the full distribution of these taxa. These latitudes result in median sea surface temperatures of 20^o^ for the low latitudes and 2^o^ for the higher latitudes for the shallow-water taxa (table 1). The variability in palaeography has other reconstructions putting the White Sea localities also at high latitudes, and Namibian localities at mid-latitudes, so would impact our analyses through different environmental temperatures [36]. To mitigate against this uncertainty, we calculate the lifespans and evolutionary rates with a range of temperatures for all taxa, so would incorporate the full range of possible environments. There is much less variation in deeper water temperatures, taken here to be greater than 1000 m, because due to density differences in water at different temperatures, bottom water is very consistently 4^o^ and −2^o^ in polar regions [37]. In order to quantify the impact of temperature on the lifespan on Ediacaran organisms, we first calculated the lifespans at the key temperatures of −2^o^, 2^o^, 4^o^, 10^o^, 20^o^ and 27°C corresponding to minimum, maximum and median environmental temperatures for organisms varying between 0.01 and 1000 g.

**Table 1.** Parameters used in the volume calculations for a range of Ediacaran taxa. Maximum reported values and ranges used. Volume refers to whether the biovolumes are calculated using a (1) cylinder, (2) dome, (3) frond shape and (4) bag or sack shape.

taxon	assemblage	length (cm)	width (cm)	depth (cm)	stem width (cm)	stem length (cm)	disc radius (cm)	disc depth (cm)	volume	habitat depth	habitat temp (^o^C)
*Dickinsonia*	White Sea	140.55	130.11	0.47	0	0	0	0	1	shallow	20
*Kimberella*	White Sea	9.36	3.22	4.00	0	0	0	0	1	shallow	20
*Trepassia*	Avalon	185.00	8.00	1.00	0	0	0	0	3	deep	4
*Tribrachidium*	White Sea	3.00	3.00	0.30	0	0	0	0	2	shallow	20
*Charnia*	Avalon	57.34	8.50	1.00	0	0	0	0	3	deep	4
*Charnia *shallow	White Sea	57.34	8.50	1.00	0	0	0	0	3	shallow	20
*Charnia *polar	Avalon	57.34	8.50	1.00	0	0	0	0	3	deep	−2
*Ernietta*	Nama	12.00	5.50	5.50	0.1	0	0	0	4	shallow	20
*Avalofractus*	Avalon	4.00	2.00	1.00	0	0	0	0	3	deep	4
*Primocandelabrum*	Avalon	32.9	75.41	1.00	5.26	32	19.5	0.5	3	deep	4
*Charniodiscus*	Avalon	30.37	7.85	1.00	2.33	7.04	3.25	1	3	deep	4
*Fractofusus*	Avalon	50.45	57.17	7.15	0	0	0	0	3	deep	4

We then calculated the lifespan for 10 iconic Ediacaran taxa (table 1). Studies of the MTE use the wet weight of organisms [13,28,38]. We use these parameters alongside the dimensions from the largest specimens for each species to infer the maximum lifespan for key Ediacaran taxa (table 1). In table 1, the dimensions are taken from the following publications: *Dickinsonia* [39,40,41] *Kimberella* [42], *Trepassia* [43], *Tribrachidium* [44,45], *Ernietta* [46], *Charnia* and *Charniodiscus* [47] and *Fractofusus* and *Primocandelabrum* [48], and then the biovolume is calculated based on gross shape and so calculated as a cylinder (*Dickinsonia*, *Kimberella*), dome (*Tribrachidium*), frond shape (*Trepassia*, *Charnia*, *Charniodiscus*, *Primocandelabrum*, *Fractofusus* and *Avalofractus*) or a bag shape (*Ernietta*). Where specimen depths are not directly measurable, they are inferred by the preserved relief and taxa (cf. [49]). Biovolume was converted to biomass as the density of seawater because aquatic organisms have a mean density of their liquid environment [50], which for deep-water organisms (i.e. below the ocean pycnocline) is 1028 g m^−3^, above which the density decreases to 1.025 kg m^−3^ [51]. To account for fossil compression, we selected specimens from localities that had undergone limited compression where possible (*Dickinsonia* and *Tribrachidium* [44], *Charnia* [20] and *Ernietta* [52]) with other taxa reconstructed following previous work (*Fractofusus* [53,54] and *Kimberella* [55]). Frondose taxa were reconstructed such that their stems had a circular cross section, similar to modern octocorals (*Avalofractus, Primocandelabrum, Charniodiscus* and *Trepassia*). There have been suggestions that rangeomorph taxa such as *Charnia* are bag-like with a thin envelope [21]. While the lack of tears or shrinkage in such taxa across tens of thousands of specimens [48] makes cell-width outer linings unlikely, in order to understand the impact of different proportions of internal cavities, i.e. different proportions of internal water percentage, for *Charnia* (as the taxa with the widest temporal and biogeographical range) we calculated how the lifespan varies across a range of percentage volume to mass ratios. To understand the impact of modularity, we calculated lifespan and evolutionary rates for Ediacaran organisms, the theoretical (−0.25), modular (−0.21) and observed unitary (−0.35) values, with the unitary values taken from the same study as the modular values to enable direct comparison.

## 3. Results

**Figure 1.**
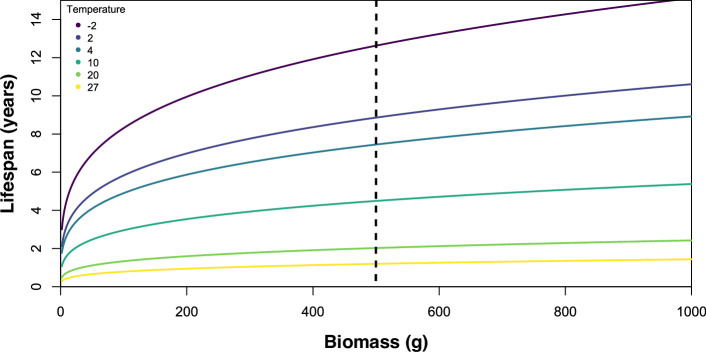
Variation of lifespan (*y*-axis) with biomass (*x*-axis) at a range of different environmental temperatures going from the hottest (27°C, yellow) to the coldest (−2°C, dark blue). Lifespans are shortest in warmer environments and for smaller organisms (left-hand side), with the greatest increase in lifespan with biomass increase seen in colder environments.

Temperature had a notable impact on lifespans as seen when lifespan was calculated from a range of biomasses (1−1000 g) (figure 1), over a range of temperatures from deep-water high latitude temperatures (−2°C) to shallow-water low latitudes (27^o^C). Colder temperatures corresponding to longer lifespans, such that for a 1 kg organism, the lifespan would be 1.33 years at 27°C versus 19.6 years at −2°C. This environmental dependence in lifespan is well known, for example, octopods have a lifespan of 0.59 years at 25.6°C versus 13 years at 1°C for similarly sized taxa [56]. In this study, we found that the absolute differences were smaller for smaller biomasses (corresponding to equation (2.2)), such that a 10 g organism lifespan would be 0.40 years at 27°C versus 5.90 years at −2°C. For *Charnia,* the lifespan ranges from 1.11 to 5.02 to 16.33 years. Changing the percentage of biovolume that corresponds to biomass acts to move the lifespan reading left along the *x*-axis, such that at 50% active biovolume at 501 g (which corresponds to *Charnia,* table 1), lifespan reduces by 16.5%, corresponding to 0.92 years at the maximum cold temperature (−2°C) or 7.49 at the modal (10°C) sea surface temperature.

**Figure 2.**
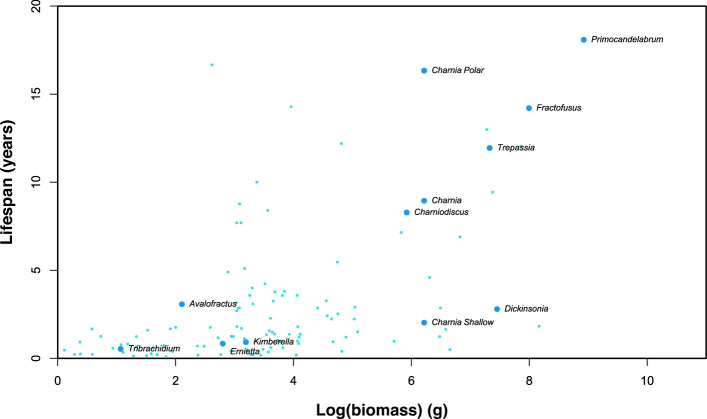
Lifespans for the 10 different Ediacaran taxa in this study (labelled, dark blue), showing three different lifespans for *Charnia* corresponding to the three different environments. Pale blue dots are the observed values for invertebrate species from [13].

For 10 different Ediacaran species, we see a range of different maximum lifespans (figure 2), from *Tribrachidium* at 0.53 years to *Primocandelabrum* at 30.2 years. A *Charnia* in the deep-water, high-latitude Avalon assemblage had a maximum lifespan of 16.34 years, in contrast to only 2.02 years for low-latitude, shallow-water *Charnia*. The largest shallow-water taxon, *Dickinsonia,* which can reach over a metre in length [57], had a maximum lifespan of 2.79 years, which emphasizes how the enviornmental temperature plays a much greater role than biomass for lifespans.

**Figure 3.**
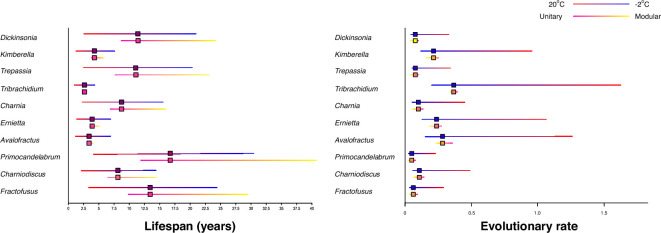
Lifespan Ediacaran organisms ranges from 0.53 years for unitary *Tribrachidium* to 40.4 years for a modular *Primocandelabrum*, influenced by different temperatures (red 20°C to blue −2°C) and modularities (yellow) and unitary (pink).

Lifespan between the deep-water, polar environment (−2°C) and the shallow-water, low-latitude environment (20°C) changed substantially (806%) (figure 3), with the largest organsims showing the largest absolute range of (*Primocandelabrum* has 26.46 years difference) while the smallest, *Tribrachdium* only varies by 3.71 years, although these organisms are not found in the same temperature ranges. The greater the biomass of an organism, the higher the impact of the modular versus unitary sensitvity (equation (2.1)), with the largest absolute difference of 28.81 and 0.35 years, respectively (349%), whereas the smallest biomass organism varied only 116% (figure 3). This increased lifetime is suggest to be because colonial organisms can have more efficient growth strategies and can survive death of individual modules [58,59]. These percentage changes were the same for evolutionary rates as they were on lifespans. Taxa similar to *Charniodiscus*, namely, *Arborea,* are suggested to be colonial [18], and so likely had the longer inferred maximum lifespans (figure 3).

## 4. Discussion

The maximum lifespans of Ediacaran organisms are strongly dependent on the environmental temperature which they inhabit, an effect amplified by their biomass. Our sensitivity analyses show that altering the percentage of biomass (<50%) does not affect maximum lifespans significantly (figure 1), providing a robustness to our estimates. We use the largest known specimens across hundreds of surfaces from Newfoundland, Canada, Charnwood Forest, UK, and Nilpena, South Australia [47,60–62], reducing the chance of significantly larger specimens being found, and the chance of the biomass estimates (so maximum lifespans) would increase from finding new specimens. Taken together, our sensitivity analyses and ranges provide a relatively robust framework for estimating the maximum lifespans of these Ediacaran organisms. Moreover our analyses are corroborated by the fact that our lifespan ranges are similar to those inferred using electronic length-frequency analysis for the Ediacaran taxon *Parvancorina* [63]; however, this alternative analysis is limited, as a single population may not be representative, so cannot constrain the maximum lifespan of Ediacaran taxa and because it does not directly include temperature dependency so their conclusions are more limited in scope.

The lifespans of animals can markedly change ecosystem carbon dynamics by organic carbon processing [64] and by driving biogeochemical cycling through their decomposition [65]. The longer lifespan, the more organic carbon (biomass) is released to the environment when it dies, thus imparting an influx of organic carbon. Coloniality, however, does not affect biomass influx in terms of lifespans since the total organic carbon released stays the same. A key driver for the selection pressure shaping the evolution of sensory organs in early animals is suggested to be the patchy resource distribution formed by the decay of animal remains of sessile taxa [2,66]. This theory is supported by increasing importance of substrate heterogeneities to Ediacaran organisms through time [67,68]. Large (i.e. long-lived) organisms increase the relative patchiness of this resource, as well as increasing the rewards of finding such patches. Smaller organisms with the same total amount of biomass would impart the same total amount of carbon, but provide less heterogeneity. Insights into adaptability are provided by evolutionary rates, which are much slower in colder environments, where the largest Ediacaran organisms are found (figure 3). As such, the capacity for organisms to fully utilize the resources provided by the larger, longer-lived organisms is reduced.

In contrast, the smaller Ediacaran taxa that inhabit the warmer low latitude, shallow-water environments, such as *Tribrachidium* and *Kimberella,* show some of the fastest evolutionary rates (figure 3), due to their relatively small body sizes coupled with their warm environmental temperatures. In extant organisms, the key downside to small body size is the predation risk [69], yet this factor can be discounted as macro-predators are not found in the Ediacaran until just before the Cambrian [70]. Therefore, the benefits of small body size, such as fast lifespans and increased evolutionary rates, as we infer in our analyses, could in part explain the large increase in morphological and taxonomic diversity that is found in the younger, warmer shallow-water Ediacaran sites [61,71,72].

## Data Availability

All input data and code are uploaded as electronic supplementary material. Supplementary material is available online [73].
